# DBA/2J Genetic Background Exacerbates Spontaneous Lethal Seizures but Lessens Amyloid Deposition in a Mouse Model of Alzheimer’s Disease

**DOI:** 10.1371/journal.pone.0125897

**Published:** 2015-05-01

**Authors:** Harriet M. Jackson, Kristen D. Onos, Keating W. Pepper, Leah C. Graham, Ellen C. Akeson, Candice Byers, Laura G. Reinholdt, Wayne N. Frankel, Gareth R. Howell

**Affiliations:** 1 The Jackson Laboratory, Bar Harbor, Maine, United States of America; 2 Sackler School of Medicine, Tufts University, Boston, United States of America; University of S. Florida College of Medicine, UNITED STATES

## Abstract

Alzheimer’s disease (AD) is a leading cause of dementia in the elderly and is characterized by amyloid plaques, neurofibrillary tangles (NFTs) and neuronal dysfunction. Early onset AD (EOAD) is commonly caused by mutations in amyloid precursor protein (APP) or genes involved in the processing of APP including the presenilins (e.g. PSEN1 or PSEN2). In general, mouse models relevant to EOAD recapitulate amyloidosis, show only limited amounts of NFTs and neuronal cell dysfunction and low but significant levels of seizure susceptibility. To investigate the effect of genetic background on these phenotypes, we generated *APP^swe^* and *PSEN1^de9^* transgenic mice on the seizure prone inbred strain background, DBA/2J. Previous studies show that the DBA/2J genetic background modifies plaque deposition in the presence of mutant APP but the impact of *PSEN1^de9^* has not been tested. Our study shows that DBA/2J.*APP^swe^PSEN1^de9^* mice are significantly more prone to premature lethality, likely to due to lethal seizures, compared to B6.*APP^swe^PSEN1^de9^* mice—70% of DBA/2J.*APP^swe^PSEN1^de9^* mice die between 2-3 months of age. Of the DBA/2J.*APP^swe^PSEN1^de9^* mice that survived to 6 months of age, plaque deposition was greatly reduced compared to age-matched B6.*APP^swe^PSEN1^de9^* mice. The reduction in plaque deposition appears to be independent of microglia numbers, reactive astrocytosis and complement C5 activity.

## Introduction

With an ever-increasing aging population, age-related neurodegenerative diseases are on the rise. Alzheimer’s disease (AD) is currently the leading cause of dementia, with the incidence only set to increase[1,2], and treatments proving unsuccessful[3–8]. Typical characteristics of AD are the presence of amyloid-β (Aβ) plaques and neurofibrillary tangles (NFTs) that lead to neuronal cell loss and cognitive deficits. Early-onset AD (EOAD, also known as familial AD) is caused by mutations in amyloid precursor protein (APP) or enzymes known to cleave APP, such as the γ-secretase, presenilin-1 (PSEN1). APP can be cleaved into multiple forms including non-amyloidogeneic (non-AD causing) and amyloidogenic (AD causing) forms [9–14]. In general, AD-relevant mutations drive APP processing towards the amyloidogenic pathway leading to the accumulation of Aβ peptides and oligomers, an early step in neuronal dysfunction, although the exact mechanisms by which APP misprocessing leads to AD are not fully understood.

Commonly used mouse models for EOAD are based on the overexpression of mutant forms of human APP, presenilins or a combination of both [10,15]. These models show a varying array of AD hallmarks, mainly related to amyloidogenesis, but do not generally recapitulate all the key AD hallmarks including NFTs, significant neuronal cell loss and the severe levels of cognitive decline observed in human patients [10,15]. These limitations have severely hampered our understanding of the mechanisms by which mutations in APP, or APP processing genes, lead to NFTs and neuronal cell loss. Therefore, to better understand the pathogenesis of AD, studies have focused on both candidate gene approaches, such as modifying certain genes and pathways or unbiased approaches where genetic background in mice carrying AD-relevant genes has been varied. The majority of mouse models are maintained on the C57BL/6J (B6) genetic background, but studies have shown that AD-relevant features can be modified by genetic backgrounds such as DBA/2J, A/J, FVB/N 129SvEvTac, 129S1 and C3H [15,16].

The DBA/2J (D2) strain is of particular interest because of its susceptibility to disorders that involve neuronal cell loss including glaucoma [17–20] and hearing loss[21,22]. It is also known to show greater levels of APP expression than B6 mice [23]. Previous studies have shown that amyloidogenesis was significantly reduced in D2 mice carrying the R140 transgene and these quantitative differences were attributed in part to genetic variation between B6 and D2 located on mouse chromosomes 1, 2 and 7 [24]. The region of mouse chromosome 2 includes a D2-specific variation that renders the complement component C5 (or Hc gene) non-functional. Mutations in complement components have previously been shown to modulate neurodegenerative disease such as Alzheimer’s disease and glaucoma [18,25–27]. Furthermore, a transcriptomics study that included the APP-Tg model on a DBA/2J genetic background identified Kinesin light chain 1 (*Klc-1*) as a modifier of Aβ [28].

AD models are known to show increased levels of spontaneous seizures but it is not clear if this is due to increased expression of APP, and therefore not necessarily relevant to human AD, or due to increased accumulation of certain cleaved forms of APP and therefore directly relevant to human AD. Recent studies suggest that seizures may be a critical part of AD pathogenesis [29–31] leading to the possibility that the increase in seizures observed in AD mouse models may not simply be a side effect of overexpression of APP. Therefore, here we assessed the impact of the DBA/2J genetic background in combination with mutant forms of APP and PSEN1 [10,32] (herein referred to as D2.*APB* ^*Tg*^) on plaque deposition. The DBA/2J genetic background is also of particular interest because of the high susceptibility to induced [33–35] or spontaneous seizures compared to other inbred strains including B6 [36]. D2.*APB* ^*Tg*^ mice were significantly more susceptible to lethal seizures at 2–3 months of age compared to B6.*APB* ^*Tg*^ mice. However, as had been shown previously in AD models that overexpress only mutant APP, D2.*APB* ^*Tg*^ mice still show a great reduction in Aβ deposition compared to B6.*APB* ^*Tg*^ mice, despite the overexpression of mutant PSEN1.

## Materials and Methods

### Mouse strains and husbandry

C57BL/6J.*APP* ^*swe*^ *Psen1* ^*de9*^ mice (MMRRC-JAX stock #034832 [10,32] referred to as B6.APB^*Tg*^) were developed by Dr. David Borchelt, and obtained from the Mutant Mouse Resource and Research Center (MMRRC) at The Jackson Laboratory. To generate cohorts, B6.*APB* ^*Tg*^ mice were mated to C57BL/6J (B6, JAX stock #000664) mice to generate both B6.*APB* ^*Tg*^ and B6 mice. To generate D2.APB^*Tg*^ mice, B6.*APB* ^*Tg*^ mice were backcrossed to DBA/2J (D2, JAX stock #000671) mice for at least 6 generations. To generate D2.*APB* ^Tg^.C5^B6^ mice, D2.*APB* ^Tg^ mice were crossed to D2.C5^B6^ mice that have been previously described [18]. All mice were maintained on a 12/12 hours (hrs) light/dark cycle. Mice were housed in 6 inch duplex wean cages with pine shavings and group-housed dependent on sex at wean. B6 and B6.*APB* ^*Tg*^ mice were maintained on LabDiet 5K54 and D2, D2.*APB* ^Tg^ and D2.*APB* ^Tg^.C5^B6^ mice were maintained on LabDiet 5K52. The Animal Care and Use Committee at The Jackson Laboratory experimentally approved all mice used in this study. Daily monitoring of mice via routine health care checks was carried out to determine general well being. This included a visual inspection of their appearance particularly the health of their coat and their weight and their activity to make sure they were not distressed. Any mice considered to be unhealthy being euthanized with IACUC approved CO_2_ euthanasia methods.

### Transgene localization by DNA FISH and sequencing

High throughput sequencing of mate pair libraries and analysis was performed as previously described [37]. For DNA FISH, mitotic chromosome spreads were prepared from the peripheral blood of B6.*APB* ^*Tg*^ mice using established methods (http://www.jax.org/cyto/blood_preps.html). Briefly, retro-orbital blood was collected into heparin and cultured with lipopolysaccharide (LPS) and phytohaemagglutin (PHA) to stimulate B and T lymphocytes, respectively, in RPMI medium supplemented with fetal bovine serum at 37C for 41–43 hours. Colchicine (5 μg/ml) was added for 20 min. to induce mitotic arrest and mitotic cells were harvested, treated in a hypotonic solution (0.56% KCl, 15 min.) and fixed ice cold 3:1, methanol:glacial acetic acid (2 × 30 mins.). Chromosome spreads were prepared by applying the suspension, drop-wise, onto the surface of clean microscope slides. DNA FISH probes were prepared from cloned *App* ^*swe*^ and *PSEN1* ^*dE9*^ cDNA sequences provided by the Borchelt laboratory. Probes were differentially labeled with biotin and DIG using nick translation according to the manufacturer’s protocol (Roche Applied Science, #10976776001) and co-hybridized to chromosome spreads using standard DNA FISH protocols [38].

### Genotyping

**Fig 1:**
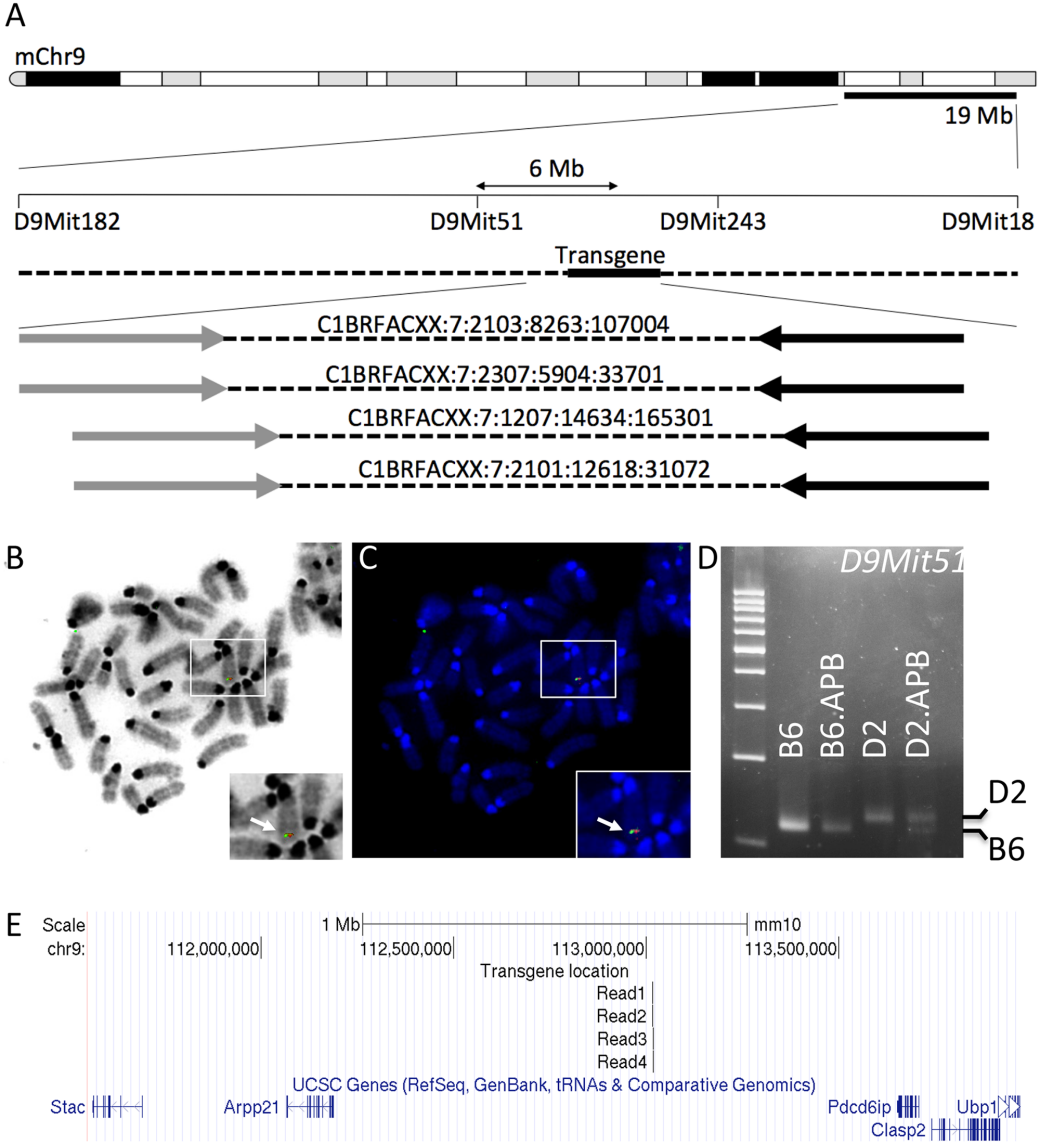
Localizing the *APB* ^*Tg*^ insertion site. (**A-C**) High throughput sequencing combined with DNA FISH localized the insertion site to a region of mouse chromosome 9. At least four paired end reads (arrows linked by dotted lines; forward reads indicated with grey arrows, reverse reads indicated by black arrows) were identified where the forward read mapped to a region of chromosome 9 and the reverse read mapped the *APP* ^*swe*^ transgene sequence (A). Fluorescent probes generated from plasmids containing both the *APP* ^*swe*^ and *PSEN1* ^*de9*^ transgenes colocalized to distal chromosome 9 (B, C) with chromosome 9 being identified due to its characteristic banding pattern. (**D**) Further confirmation of the transgene localization was obtained using markers polymorphic between B6 and D2. *D9Mit51* lies approximately 6 Mb upstream of the transgene insertion site (A) and D2.*APB* ^*Tg*^ mice retain B6 sequence for *D9Mit51* in this region despite having backcrossed to D2 for 6 generations. (**E**) The insertion site (identified by localizing the four forward reads (shown in A) using BLAT at the UCSC genome browser) lies is a relative gene desert flanked by the *Arpp21* and *Pdcd6ip* genes. Coordinates are from the GRCm38/mm10 mouse genome build.

Standard genotyping protocols were followed to confirm the presence of the *APP* ^*swe*^/*PSEN1* ^*dE9*^ transgenes (see http://jaxmice.jax.org/strain/005864). Once the insertion site for the transgene was located via next generation sequencing and FISH (Fig 1), polymorphic markers were identified using a combination of Ensembl and Mouse Genome Informatics. Polymorphic markers included D9Mit182 (D2 = 117bp, B6 = 99bp), D9Mit51 (D2 = 134bp, B6 = 124bp), D9Mit243 (D2 = 117bp, B6 = 95bp) and D9Mit18 (D2 = 204bp, B6 = 180bp). DNA was amplified by PCR from B6, B6.APB^Tg^, D2 and D2.APB^Tg^ under standard conditions. To detect the selected region from Chromosome 2 that included C5, polymorphic marker D2MIT367 (D2 = 162bp, B6 = 146bp) was used, as previously described [18]. All products were then resolved in a 5% gel.

### Electroconvulsive threshold test

The methods used here are the same as described in Sun et al., 2013 [39]. In brief, mice were restrained by hand and drops containing 0.5% tetracaine and 0.9% NaCl were applied to each eye. Electrical current was applied through silver transcorneal electrodes using a pulse generator (Ugo Basile model 7801). Square wave pulses were delivered with the following parameters: 280 or 299 Hz, 0.2 s duration, 1.6 ms width and variable current between 4 and 8 mA. Testing was started at a current estimated to be just below or at the expected mean generalized seizure threshold and responses were scored by the following scale: 0—animal walks away with no sign of disruption; 1-animal freezes briefly, then walks away; 2- very brief jaw and forelimb clonus; 3- prolonged jaw and forelimb clonus including loss of posture of several seconds (minimal generalized seizure). Stimulation was applied once daily using a staircase approach until the threshold to a minimal generalized seizure was determined. Mice were euthanized via CO_2_ methods after experimentation. Cohorts consisted of males and females from D2 and D2.*APB* ^Tg^ (n = D2: F = 6 M = 6, D2.*APB* ^Tg^: F = 3, M = 6)

### Seizure monitoring

Seizure rates were determined during routine health care checks, by whole colony monitoring, and seizure events were determined by the characteristic clenched forelimbs and stretched out hind limbs. No evidence of sub-lethal seizures was observed although long term video monitoring was not performed and so this cannot be ruled out. To reduce the impact of environmental factors on seizure incidence, all mice were housed under ‘quiet’ conditions where noise was kept to a minimum. Seizure lethality cohorts were as follows: Female; B6 = 148, B6.*APB* ^Tg^ = 128, D2 = 32, D2.*APB* ^Tg^ = 92. Male; B6 = 168, B6.*APB* ^Tg^ = 150, D2 = 26, D2.*APB* ^Tg^ = 80. Upon completion of the study, mice were humanely euthanized using CO_2_.

### Tissue harvesting, protein isolation and sectioning

Mice were administered a lethal dose of Ketamine/Xylazine intraperitoneal injection, in accordance to IACUC protocols, and transcardially perfused with 1xPBS at 6 months of age. Brains were then dissected, the right hemisphere was snap frozen for protein isolation and the left hemisphere was fixed in 4% paraformaldehyde overnight at 4°C, rinsed with 1xPBS, cryoprotected in 10% and 30% sucrose at 4°C then embedded in OCT. Frozen brains were sectioned at 25μm and stored at -80°C until required. Protein was extracted with Trizol Reagent (Life Technologies cat#15596–018) following manufacturer’s guidelines. Pellets were resuspended in a solution of 1:1 8M urea and 1% SDS.

### Immunofluorescence, Thioflavin S staining, and image capture

Sections were incubated overnight at 4°C in the following primary antibodies: chicken polyclonal anti-GFAP (1:250, Acris Antibodies); rabbit polyclonal anti-IBA1 (1:250, Wako); rabbit polyclonal anti NeuN (1:300, Cell Signaling Inc) and mouse polyclonal anti-AT8 (1:100, Pierce Antibodies). All antibodies were diluted in PBTB (1xPBS, 1% TritonX-100 and 1%BSA) containing 10% normal goat serum. After primary incubation, sections were washed 3 times in PBT (1xPBS with 1% TritonX-100) and incubated with their respective secondary antibody (goat anti-chicken Alexa Fluor 488, goat anti-rabbit Alexa Fluor 488/594, goat anti-mouse Alexa Fluor 488, 1:1000 dilution, Life Technologies) for 2hrs at room temperature. All sections were then counterstained with DAPI and mounted with Aqua PolyMount. For Thioflavin S staining, sections stained with IBA1 and GFAP were further counterstained with 1% Thioflavin S (diluted in a 1:1 water:ethanol ratio). Slides were incubated for 8 minutes (mins) at room temperature in 1% Thioflavin-S, washed in 80% ethanol, then 95% ethanol and finally in dH2O and mounted. Images were taken using either the Leica SP5 confocal microscope or the Zeiss Axio Imager.Z2. Quantification of cell numbers was performed as follows. For plaque counts, the number of plaques present in the entire cortical region from a central section for each mouse was determined. For IBA1^+^ cells, 3 equally spaced images were captured (using 20x optical lens) of the cortex from a central section of each mouse. For NeuN^+^ cells, 3 equally spaced images were captured (using 20x optical lens). For IBA1^+^ surrounding plaques, images of 6 plaques per brain were imaged (using 20x optical lens). Images were processed and cells counted using the cell counter plugin for ImageJ/FIJI. Cells from the 3 images from each mouse were totaled and then averaged across mice.

### Western Blotting

Protein sample concentration was determined via DC assay (Bio-Rad) and diluted to 1.5μg total protein. Samples were heated to 95°C for 5mins then loaded onto a 12% TGX stain free gel (Bio-Rad). Gels were run at 150V for 45mins and then transferred to nitrocellulose membrane (Life Technologies) via the iBlot for 7mins. Blots were then incubated for 2 nights in 5% skim milk powder block in 0.1% PBS-Tween with 6E10 antibody (1:2000, Covance/BioLegend) at 4°C. Blots were then washed 3 times in 0.1% PBS-Tween and incubated in the appropriate secondary antibody (Anti-Mouse IgG 1:30,000, Millipore) for 2hrs at room temperature. Detection was carried out using ECL detection reagents (GE Healthcare). Blots were treated with 0.25% sodium azide, and thoroughly washed, then further probed with anti-beta actin (1:1000, Abcam) in 0.1% PBS-Tween overnight at 4°C, washed 3 times and incubated with secondary antibody (Anti-mouse IgG 1:30,000, Millipore) for 2hrs at room temperature, washed and detected.

### ELISA

Aβ42 levels were determined using the Life Technologies detection kit (cat#KHB3442) following the specified instructions. Protein samples were diluted 1:50 in standard diluent buffer to ensure that the levels of Urea and SDS were compatible with the kit. Samples were then compared to a standard curve and Aβ42 concentrations were established against the samples protein concentration.

### Statistical tests

At least four female mice were assessed for each group. Statistical significance was determined by ANOVA and student t-tests, performed using GraphPad or Excel.

## Results

### APP^swe^ and PSEN1^de9^ transgene insertion sites are located on distal chromosome 9

B6.*APB* ^*Tg*^ mice contain two transgenes, *APP* ^*swe*^ and *PSEN1* ^*de9*^ [10,32] that are closely linked but the chromosomal integration sites of the transgenes have not been shown. For backcrossing Therefore, paired-end high throughput DNA sequencing of DNA performed from B6.*APB* ^*Tg*^ mice and aligned reads to the mouse genome (Fig 1). Four paired-end reads were identified where the forward reads mapped to mouse chromosome 9 (at approximately 113 Mb, GRCm38) and the reverse reads mapped to the APP sequence from the transgene. Fluorescent *in situ* hybridization using probes for the *APP* ^*swe*^ and *PSEN1* ^*de9*^ transgenes confirmed the distal mouse chromosome 9 integration site (see methods and Fig 1). Furthermore, co-segregation analysis of the B6 derived transgene locus and D9*Mit51*, a DNA marker that is polymorphic between B6 and D2 also implicated distal chromosome 9. Assessment of the fragment sizes of *D9Mit51* amplified from D2.*APB* ^*Tg*^ showed a fragment size similar to that seen in B6.*APB* ^*Tg*^ and B6 mice but different from D2 mice (see Fig 1). This is consistent with the region flanking the insertion site originating from the B6 genetic background in D2.*APB* ^*Tg*^ mice. Collectively, these data support integration of the *APP* ^*swe*^ and *PSEN1* ^*de9*^ transgenes at approximately 113 Mb on mouse chromosome 9, approximately 600 kb distal to the *Arpp1* gene and approximately 400 kb proximal to the *Pdcd6ip* gene.

### Increased spontaneous lethal seizures in D2.APB^Tg^ mice compared to B6.APB^Tg^ mice

**Fig 2:**
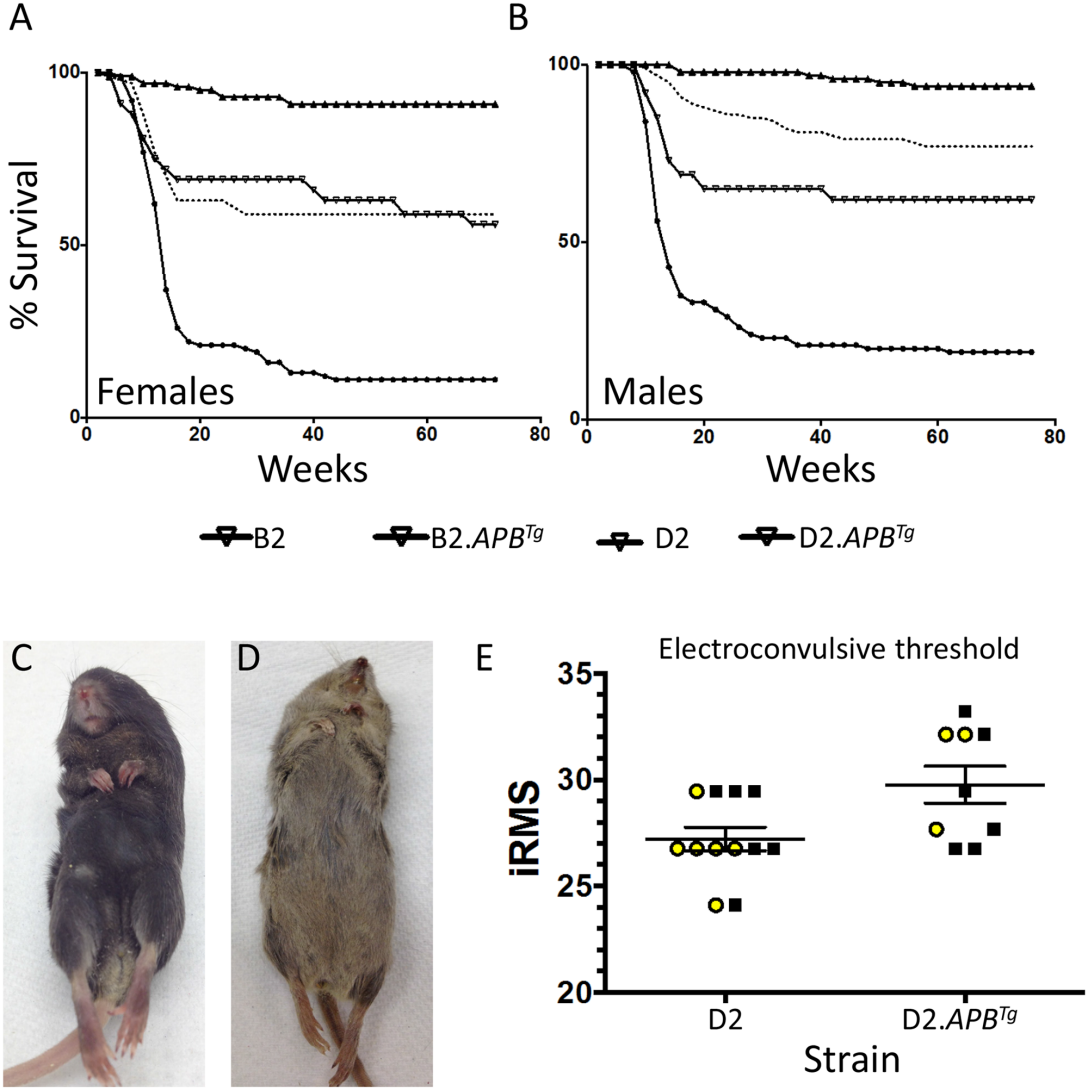
D2.*APB* ^*Tg*^ mice are more susceptible to lethal seizures compared to B6.*APB* ^*Tg*^ mice. (**A, B**) Kaplan Meier curves showing the rate of background dependent death between the 4 cohorts of mice (B6, B6.*APB* ^*Tg*^, D2 and D2.*APB* ^*Tg*^). Both female (A) and male (B) D2.*APB* ^*Tg*^ mice carrying the transgenic region are highly susceptible to premature death. D2 mice show a significant increase in premature death compared to B6 mice. (**C, D**) B6.*APB* ^*Tg*^ (C) and D2.*APB* ^*Tg*^ mice (D) that have died prematurely show a characteristic seizure pose with clenched fore limbs and stretched out hind limbs. (**E**) Despite the increase in spontaneous lethal seizures in D2.*APB* ^*Tg*^ mice compared to D2 mice, a slight increase in electroconvulsive threshold was observed in D2.*APB* ^*Tg*^ mice compared to D2 mice (p = 0.019). Yellow circles = females, black squares = males.

To generate the D2.*APB* ^*Tg*^ strain, the *APP* ^*swe*^ */*PSEN1^de9^ transgenes were backcrossed to DBA/2J for at least 6 generations. As backcrossing reached 4 generations it was clear that D2.*APB* ^*Tg*^ mice were more susceptible to premature death compared to either wild type D2 mice or B6.*APB* ^*Tg*^ mice. To fully assess premature lethality, cohorts of B6, B6.*APB* ^*Tg*^, D2 and D2.*APB* ^*Tg*^ mice were established and aged (Fig 2). Each cohort consisted of at least 80 males and 80 females. Approximately 43.7% of D2.*APB* ^*Tg*^ males and 38% D2.*APB* ^*Tg*^ females died before 3 months of age, with only 26.3% of males and 19.6% females surviving past 7 months of age. This compared to approximately 84.7% of B6.*APB* ^*Tg*^ males and 59.4% B6.*APB* ^*Tg*^ female mice. In our colony, D2 mice showed a lower level of premature lethality (68.8% females and 65.4% of males surviving past 7 months of age). B6 mice showed little or no premature death. Interestingly, D2.*APB* ^*Tg*^ mice that survived past 6 months of age did not appear to suffer increased lethality compared to B6.*APB* ^*Tg*^ mice of the same age, suggesting that young adult D2.*APB* ^*Tg*^ mice are more prone to premature lethality than older adults.

To investigate the cause of the premature lethality, cohorts of mice were observed daily. All mice that were found dead showed the characteristic pose of a lethal seizure that consisted of clenched front limbs and stretched out hind limbs (Fig 2c and 2d). This confirms that the DBA/2J genetic background is exacerbating the spontaneous lethal seizures that are seen in AD mouse models that overexpress mutant APP. Interestingly, however, we did not observe a decrease in electroconvulsive threshold (ECT) in D2.*APB* ^*Tg*^ mice compared to D2 mice, in fact D2.APBTg mice had a slightly higher ECT compared to D2 controls. This small effect appears to be only observed in female mice and may be due to the B6-derived congenic interval on chromosome 9 in D2.*APB* ^*Tg*^ mice. Cumulatively, our data supports that the increased lethal seizure rate in D2.*APB* ^*Tg*^ mice, compared to B6.*APB* ^*Tg*^ mice, is as a result of DBA/2J-specific genetic factors.

### No overt neuronal cell loss in D2.APB^Tg^ mice at 6 months of age

**Fig 3:**
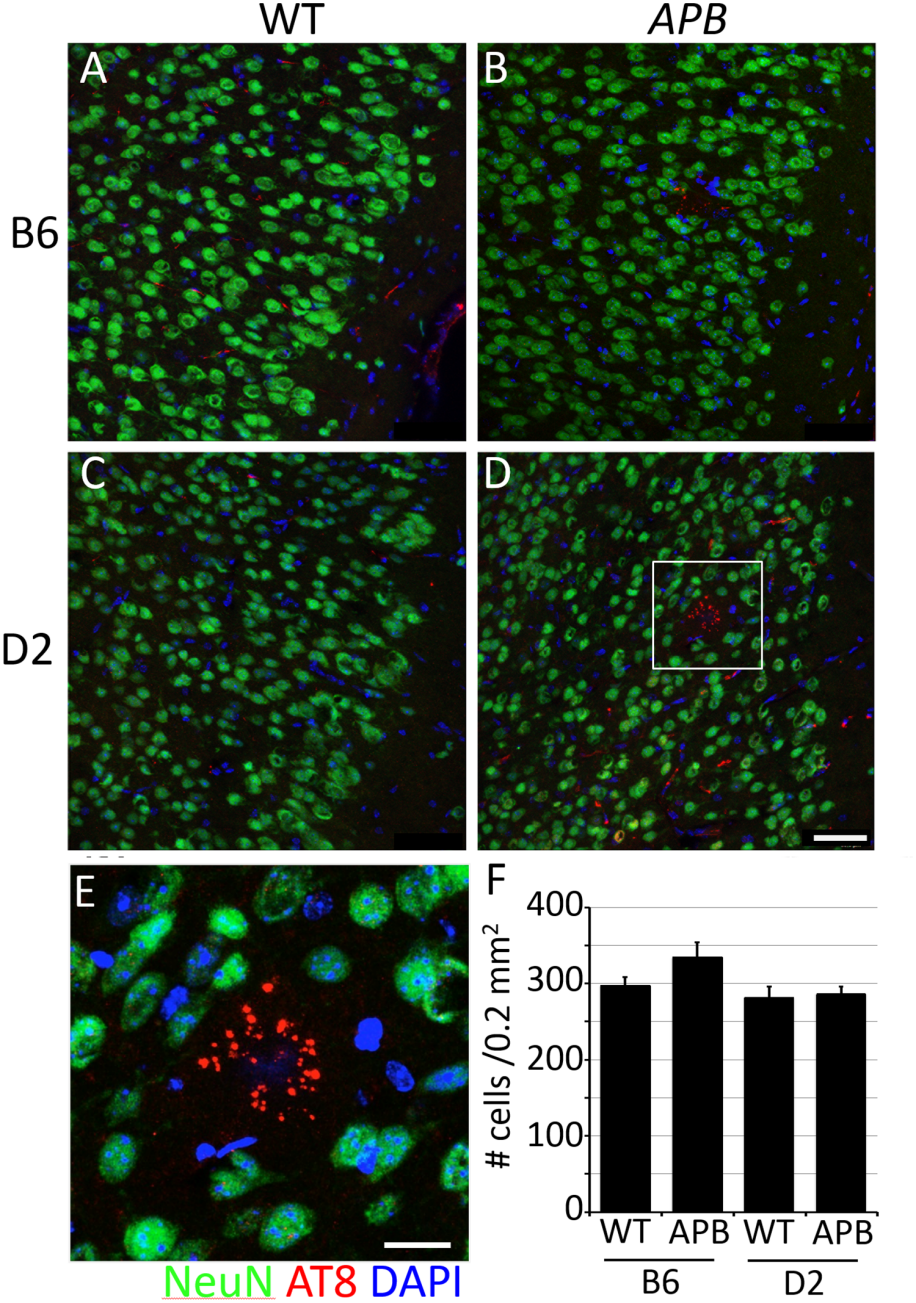
No overt changes in cortical neurons in D2.*APB* ^*Tg*^ mice. (**A-D**) NeuN+ cells were counted in B6 (A), B6.*APB* ^*Tg*^ (B), D2 (C) and D2.*APB* ^*Tg*^ (D) mice. Representative images are shown and all mice showed normal neuronal morphology in three different regions of the cortex. (E) No significant difference was observed in pTau aggregates (AT8 antibody, red) in D2.*APB* ^*Tg*^ mice (E) compared to B6.*APB* ^*Tg*^ mice. (F) NeuN+ cells were counted in each of the 4 cohorts, in 3 discrete cortical areas (4 mice per cohort). No statistically significant difference is seen between the different cohorts (p = 0.053). Scale bars: A-D = 50μm, E = 10μm.

B6.*APB* ^*Tg*^ mice begin to develop plaques at around 6 months of age in both males and females with plaque load peaking around 10–12 months of age. Minor neuronal cell loss and small cognitive deficits are only observed in older mice. Astroglial and microglial activation generally correlates with plaque deposition. Females generally show a higher degree of plaque deposition at older ages, although no obvious difference is observed at younger ages. Due to the high degree of premature death in D2.*APB* ^*Tg*^ mice it was not possible to assess AD-relevant phenotypes in mice older than 6 months of age. APP^swe^ and PSEN1^de9^ induce neuronal cell dysfunction and loss in mice in some contexts [40–43], but AD relevant features in DBA/2J mice expressing these two transgenes have not been assessed. Therefore, D2.*APB* ^*Tg*^ mice and appropriate controls (B6.*APB* ^*Tg*^, B6 and D2 mice) were aged to 6 months and assessed for neuronal cell loss using NeuN (Fig 3). Neurons were assessed in the three different regions of the cortex (see methods). No significant difference in neuron number was observed between D2 and D2.*APB* ^*Tg*^ mice at 6 months of age.

### Significant reduction in plaque deposition in D2.APB^Tg^ compared to B6.APB^Tg^ mice

**Fig 4:**
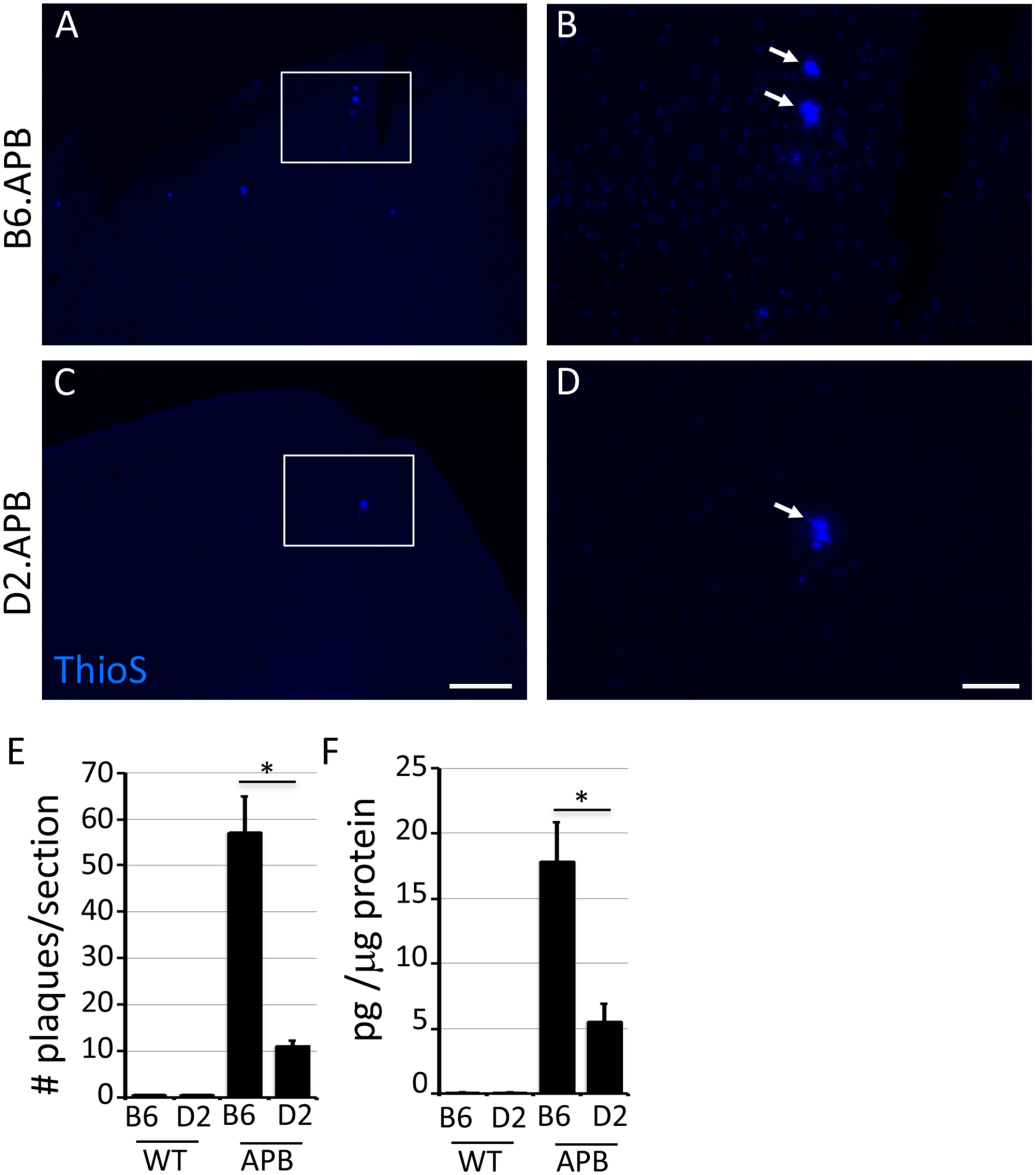
Plaque deposition is dramatically reduced in D2.*APB* ^*Tg*^ mice compared to B6.APB^*Tg*^ mice. (**A-D**) Representative images of ThioS-labeled plaques in the superior cortex from B6.*APB* ^*Tg*^ (A, B) and D2.*APB* ^*Tg*^ (C, D) mice. Boxed regions in A and C are enlarged in B and D respectively. (**F**) Plaque counts from the cortex confirm a significant reduction in plaque number in D2.*APB* ^*Tg*^ compared to B6.*APB* ^*Tg*^ mice (p = 0.001). (**E**) Aβ42 levels (by ELISA) are significantly reduced in D2.*APB* ^*Tg*^ compared to B6.*APB* ^*Tg*^ mice (p = 0.0086). Scale bars: A, B = 200μm; C-D = 50μm.

Previous reports showed that mice expressing mutant forms of APP on the D2 genetic background had lower levels of Aβ deposition compared to mice expressing mutant APP on a B6 background[16,24]. However, the impact of both mutant APP and PSEN1 in DBA/2J mice has not previously been tested. Therefore, plaque deposition in D2.*APB* ^*Tg*^ mice was assessed. Despite the presence of mutant APP and PSEN1 that both drive APP processing down the amyloidogenic pathway, there was still a significant reduction in ThioS+ plaques in the cortex of D2.*APB* ^*Tg*^ compared to B6.*APB* ^*Tg*^ mice (Fig 4A–4E). This reduction corresponded with a reduction in soluble Aβ42 levels (Fig 4F).

### Reduced plaque load in D2.APB^Tg^ mice does not correlate with increased glial activation

**Fig 5:**
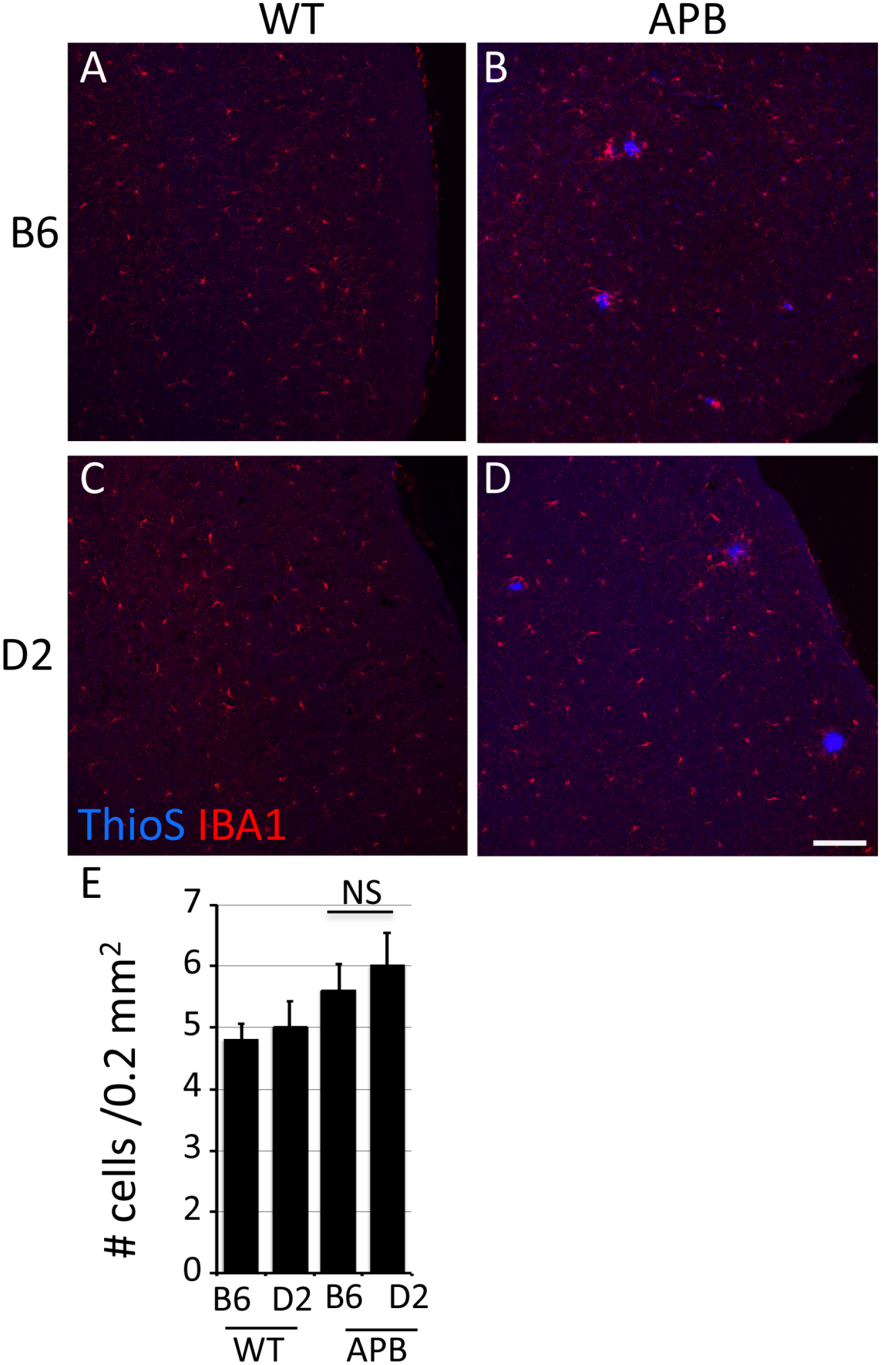
Microglia respond equally in D2.*APB* ^*Tg*^ and B6.*APB* ^*Tg*^ mice compared to wild type controls. (**A-D**) Representative images of IBA1^+^ cells in the cortex of B6 (A), B6.*APB* ^*Tg*^ (B), D2 (C) and D2.*APB* ^*Tg*^ (D) mice. (**E**) IBA1^+^ cells were counted in three different regions of the cortex in all 4 strains of mice. The levels of microglia show a mild increase in D2.*APB* ^*Tg*^ and B6.*APB* ^*Tg*^ mice compared to their wild type controls mice, but there is no significant difference microglia numbers comparing D2.*APB* ^*Tg*^ with B6.*APB* ^*Tg*^ mice. NS = not significant. Scale Bar = 100μm.

**Fig 6:**
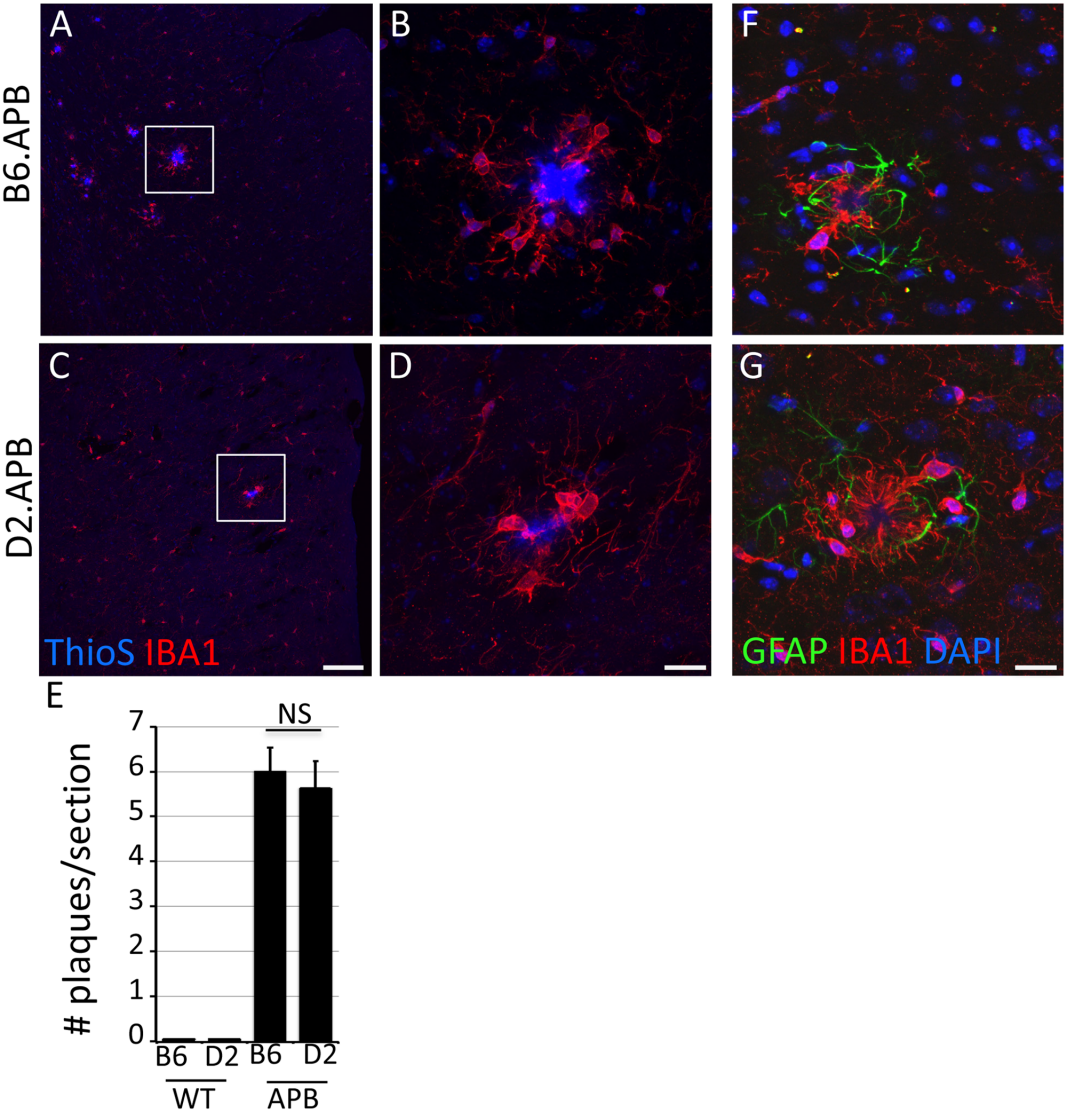
No overt difference in glial responses in plaque regions in D2.*APB* ^*Tg*^ compared to B6.*APB* ^*Tg*^ mice. (**A-D**) Representative images of IBA1^+^ cells localized to Thio S-labeled plaques in the superior cortex from B6.*APB* ^*Tg*^ (A, B) and D2.*APB* ^*Tg*^ (C, D) mice. (**E**) No significant differences were observed in the number of IBA1^+^ cells surrounding plaques in D2.*APB* ^*Tg*^ compared to B6.*APB* ^*Tg*^ mice (p = 0.65). (**F-G**) There was also no obvious difference in the level of astrocyte reactivity (judged by levels of GFAP staining) in regions of plaques in D2.*APB* ^*Tg*^ compared to B6.*APB* ^*Tg*^ mice. NS = Not significant. Scale bars: A, C = 100μm; B, D, F, G = 20μm.

Both microglia and astrocytes have been shown to aid plaque clearance and so to assess whether the reduction in plaque load in D2.*APB* ^*Tg*^ mice could be due to glial activation we first assessed IBA1^+^ microglia in the cortex of D2.*APB* ^*Tg*^ mice compared to controls (Fig 5). Both B6.*APB* ^*Tg*^ and D2.*APB* ^*Tg*^ mice showed an increase in microglial numbers compared to their wild type controls (B6 and D2 respectively). However, there was no significant difference in the number of IBA1^+^ microglia in D2.*APB* ^*Tg*^ compared to B6.*APB* ^*Tg*^ mice. We also saw no overall increase in astrocyte reactivity (judged using the intensity of glial fibrillary acidic protein, GFAP), in the cortex of D2.*APB* ^*Tg*^ mice compared to control mice. We also saw no significant difference in the number of IBA1^+^ cells surrounding plaques in D2.*APB* ^*Tg*^ and B6.*APB* ^*Tg*^ mice (Fig 6). This would suggest that low levels of plaque deposition in D2.*APB* ^*Tg*^ mice are not due to an increase in clearance of Aβ peptides by microglia or astrocytes.

### Complement C5 does not modulate AD phenotypes in D2.APB^Tg^ mice by 6 months of age

**Fig 7:**
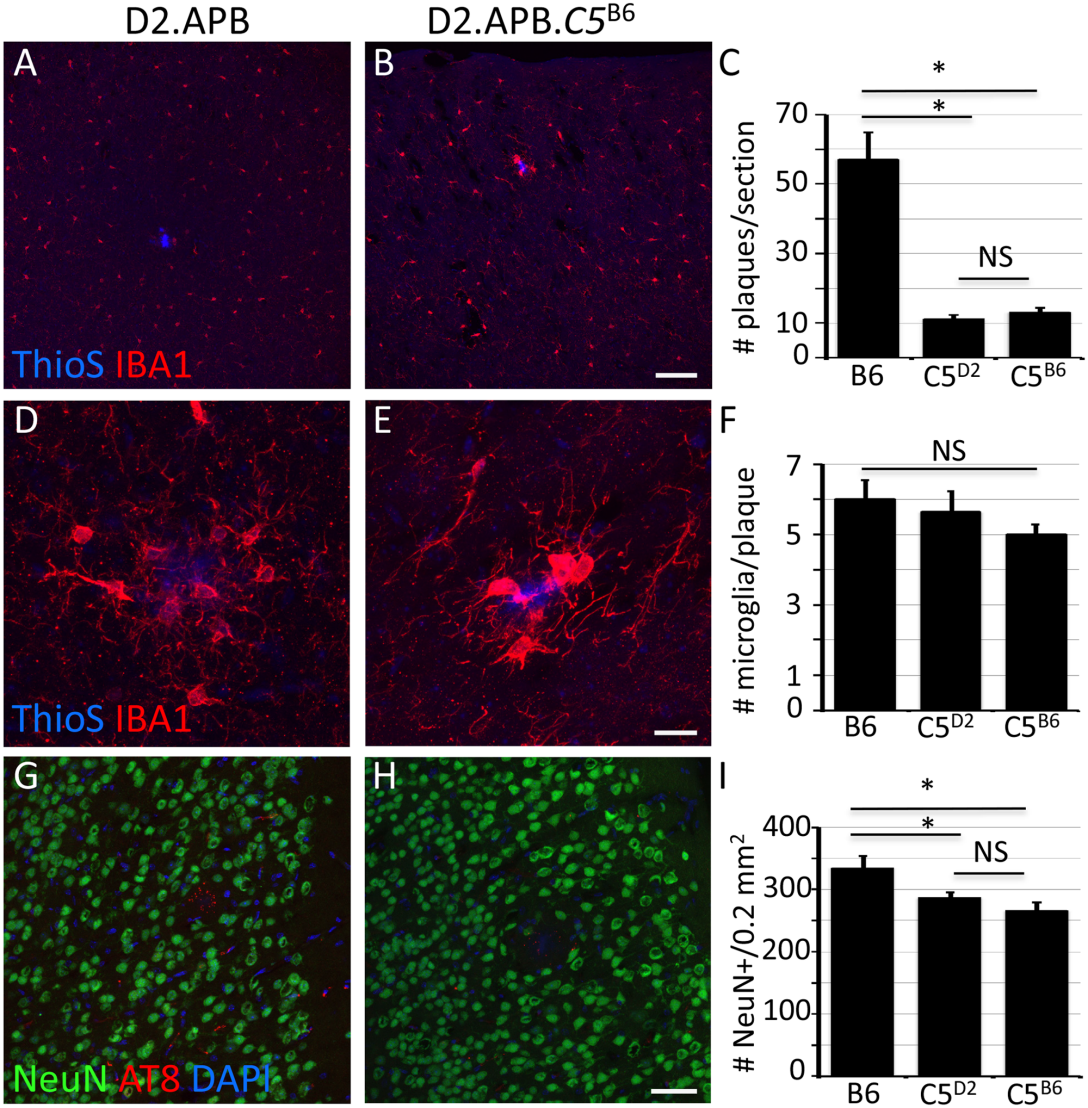
C5 sufficiency does not affect disease state in D2.*APB* ^*Tg*^ mice. (**A-C**) Plaque deposition is unchanged in D2.*APB* ^*Tg*^.*C5* ^*B6*^ (*C5* ^*B6*^) mice compared to D2.*APB* ^*Tg*^ (C5^D2^) mice (p = 0.251). Similarly to D2.*APB* ^*Tg*^ mice, D2.*APB* ^*Tg*^.*C5* ^*B6*^ show significantly less plaque deposition compared to B6.*APB* ^*Tg*^ (B6) mice (p = 0.001). (**D-F**) The number of IBA1^+^ cells surrounding plaques in not different in D2.*APB* ^*Tg*^.*C5* ^*B6*^ (*C5* ^*B6*^) mice compared to either D2.*APB* ^*Tg*^ (C5^D2^) or B6.*APB* ^*Tg*^ (B6) mice (p = 0.354 and p = 0.087 respectively). (**G-I**) No significant difference was observed in NeuN^+^ cells in the cortex in D2.*APB* ^*Tg*^.*C5* ^*B6*^ (*C5* ^*B6*^) mice compared to D2.*APB* ^*Tg*^ (C5^D2^, p = 0.44). Scale bars: A, B = 100μm; D, E = 20μm; G, H = 50μm.

Previous studies have reported that the reduction in plaque load in mouse models of AD on a D2 genetic background was due in part to gene(s) located on a region of mouse chromosome 2 [24]. This region harbors a variation that renders the complement component C5 protein non-functional and prevents the formation of the membrane attack complex (MAC). This terminal step in the complement cascade has been shown to be important in modulating phenotypes in a variety of different neurodegenerative diseases [18,27,44–48]. Therefore to determine whether C5 modulates seizure and AD-relevant phenotypes, we generated D2.*APB* ^*Tg*^ mice that carry a functional copy of *C5* (D2.*APB* ^*Tg*^.*C5* ^*B6*^, see methods, [18]). Mice were aged to 6 months and assessed for lethal seizures and AD phenotypes. C5 did not impact the level of lethal seizures, with approximately 30.4% of D2.*APB* ^*Tg*^.*C5* ^*B6*^ mice dying before 3 months of age (data not shown). For those mice that survived to 6 months of age, D2.*APB* ^*Tg*^.*C5* ^*B6*^ mice showed similar amounts of plaque deposition (assessing both overall plaque Aβ levels and number of plaques) compared to D2.*APB* ^*Tg*^ mice (Fig 7A–7E). Finally, there was no significant difference in cortical neuronal cell counts between B6.*APB* ^*Tg*^, D2.*APB* ^*Tg*^ or D2.*APB* ^*Tg*^.*C5* ^*B6*^. These data indicate that, at least at the time points assessed, C5 does not impact seizure or AD-relevant phenotypes that occur in *D2*.*APB* ^*Tg*^ mice.

## Discussion

Here, we generated D2.*APB* ^*Tg*^ mice by backcrossing the *APP* ^*swe*^ and *PSEN1* ^*de9*^ transgenes on to the D2 genetic background. Multiple models relevant to AD, including the B6.*APB* ^*Tg*^ mouse model, present an increased level of seizures compared to wild type controls [49–51]. This is postulated to be due to the overexpression of APP and not necessarily due to factors directly relevant to AD. However, the mechanisms of seizure susceptibility in AD mouse models that overexpress APP are not fully understood. This is in part due to the relatively low incidence of seizures (often less than 30%) in these strains. To our knowledge, the D2.*APB* ^*Tg*^ mouse model described here shows the highest level of lethal seizures reported in an AD mouse model. Therefore, in combination with the B6.*APB* ^*Tg*^ strain, D2.*APB* ^*Tg*^ is a complementary strain to study the increased rate of seizures in AD models. Although it is not clear why D2.*APB* ^*Tg*^ mice show this significant increase in lethal seizures compared to B6.*APB* ^*Tg*^ mice, we speculate it may be due to the fact that D2 mice are highly seizure prone. Studies have shown that they have one of the lowest thresholds for induced seizures and they also have a modest incidence of spontaneous spike-wave discharges, the electroencephalographic hallmark of absence seizures [33,34,52].

Despite the presence of mutant forms of both APP and PSEN1, plaque burden is still significantly reduced on a D2 genetic background. This observation has been seen in two AD mouse models overexpressing only mutant APP on a D2 background[16,28]. However, our study is the first to show a similar effect in the presence of mutant PSEN1. Previous studies have suggested that at least three loci on chromosomes 1, 2 and 7 harbor D2-specific variations that impact plaque deposition [24]. An additional study using a D2-specific AD model showed that the kinesin light chain-1 (*Klc1*) gene, located on chromosome 12, can significantly modulate plaque deposition [28]. This finding makes the D2 genetic background an attractive background for identifying genetic factors relevant to plaque deposition.

Mouse models relevant to AD recapitulate some features of AD, such as amyloidosis, but do not show key hallmarks such as widespread cognitive decline. These limitations have hampered the discovery of genes and pathways that can be targeted for improved therapies. Therefore, to aid in the identification of new genes and pathways it is necessary to generate alternative mouse strains relevant to AD. One approach is to adapt existing models by altering the genetic background, an approach that has proven fruitful in the identification of genetic factors that modulate other disease phenotypes [53–58]. To date only a few studies have applied this approach to AD models with some promising but as yet limited results [15,16,24,28]. In this study, we followed on from previous studies that showed that the D2 genetic background harbors genetic factors that reduce levels of plaque deposition in the presence of mutant APP. However, our model is confounded by the fact that lethal seizures are highly penetrant in D2.*APB* ^*Tg*^ mice making this model less favorable for studying later stages of AD. Additional studies are underway in our lab to assess AD relevant phenotypes in mice carrying the *APP* ^*swe*^ and *Psen1* ^*de9*^ transgenes on a variety of other genetic backgrounds including wild derived strains (such as CAST/Ei) that are most genetically diverse compared to B6 mice. Several other less common inbred mouse strains also have very low seizure thresholds [59] and it will be interesting to examine the relationship between APP processing and seizures in these strains.

Ultimately, it will be most advantageous to develop mouse models that show critical features of human AD (including plaque deposition and neuronal cell loss) in the absence of overexpressing mutant APP or mutant APP processing enzymes such as PSEN1. This is the case in the majority of human cases of AD and the development of these models will allow us to better understand genetic and environmental factors that interact to cause human AD and lead to improved treatments.

## Supporting Information

S1 Checklist (PDF)
